# Temporal proteomics of human cerebrospinal fluid after severe traumatic brain injury

**DOI:** 10.1186/s12974-022-02654-0

**Published:** 2022-12-08

**Authors:** Sandy R. Shultz, Anup D. Shah, Cheng Huang, Larissa K. Dill, Ralf B. Schittenhelm, M. Cristina Morganti-Kossmann, Bridgette D. Semple

**Affiliations:** 1 Department of Neuroscience, Monash University, Melbourne, VIC Australia; 2 Alfred Health, Prahran, VIC Australia; 3 Department of Medicine (Royal Melbourne Hospital), The University of Melbourne, Parkville, VIC Australia; 4 Health and Human Services, Vancouver Island University, Nanaimo, Canada; 5 Monash Proteomics and Metabolomics Facility, Monash University, Clayton, VIC Australia; 6 Monash Bioinformatics Platform, Monash University, Clayton, VIC Australia; 7 The Perron Institute for Neurological and Translational Science, Nedlands, WA 6009 Australia; 8 Department of Epidemiology & Preventive Medicine, Monash University, Prahran, VIC Australia; 9 Department of Child Health, Barrow Neurological Institute at Phoenix Children’s Hospital, Phoenix, AZ USA; 10 University of Arizona College of Medicine, Phoenix, AZ USA

**Keywords:** Protein, Biomarker, Inflammation, TBI, Secondary injury, Neutrophil

## Abstract

The pathophysiology of traumatic brain injury (TBI) requires further characterization to fully elucidate changes in molecular pathways. Cerebrospinal fluid (CSF) provides a rich repository of brain-associated proteins. In this retrospective observational study, we implemented high-resolution mass spectrometry to evaluate changes to the CSF proteome after severe TBI. 91 CSF samples were analyzed with mass spectrometry, collected from 16 patients with severe TBI (mean 32 yrs; 81% male) on day 0, 1, 2, 4, 7 and/or 10 post-injury (8–16 samples/timepoint) and compared to CSF obtained from 11 non-injured controls. We quantified 1152 proteins with mass spectrometry, of which approximately 80% were associated with CSF. 1083 proteins were differentially regulated after TBI compared to control samples. The most highly-upregulated proteins at each timepoint included neutrophil elastase, myeloperoxidase, cathepsin G, matrix metalloproteinase-8, and S100 calcium-binding proteins A8, A9 and A12—all proteins involved in neutrophil activation, recruitment, and degranulation. Pathway enrichment analysis confirmed the robust upregulation of proteins associated with innate immune responses. Conversely, downregulated pathways included those involved in nervous system development, and several proteins not previously identified after TBI such as testican-1 and latrophilin-1. We also identified 7 proteins (GM2A, Calsyntenin 1, FAT2, GANAB, Lumican, NPTX1, SFRP2) positively associated with an unfavorable outcome at 6 months post-injury. Together, these findings highlight the robust innate immune response that occurs after severe TBI, supporting future studies to target neutrophil-related processes. In addition, the novel proteins we identified to be differentially regulated by severe TBI warrant further investigation as potential biomarkers of brain damage or therapeutic targets.

## Background

Traumatic brain injury (TBI) can range from mild to severe and is a leading cause of death and disability worldwide [1]. Severe TBI survivors often experience a range of persisting or permanent neurological consequences such as cognitive deficits, motor dysfunction, social and emotional abnormalities, posttraumatic epilepsy, and an increased risk of neurodegenerative conditions [2, 3]. There is a knowledge gap pertaining to biomarkers that can reliably prognosticate functional outcomes in severe TBI patients, with no effective treatments available to mitigate these deficits [4]. As such, there is an urgent need to discover new biomarkers and therapeutic targets to improve the clinical management of severe TBI.

The cerebrospinal fluid (CSF) represents a repository for various molecules (e.g., proteins) released from the brain [5]. Because CSF is often accessible via extraventricular devices to monitor ICP and drain CSF in the acute and sub-acute stages of severe TBI [6], it offers a direct source of fluid to longitudinally assess the evolving pathophysiology of TBI. Several studies have examined CSF from TBI patients, with the majority taking a targeted approach measuring biomarkers related to neuronal and axonal injury, glial activation, inflammation, and/or cerebrovascular damage, precisely mirroring the molecular and cellular changes occurring in the brain in response to injury [4, 7–19].

While these studies have been informative and advanced the understanding of TBI, the scarcity of TBI biomarkers and effective treatment targets warrants further research. In the present study, we cast a wide, unbiased net by applying high-resolution mass spectrometry (LC–MS/MS) to quantify > 1000 proteins from CSF samples collected serially over 10 days post-injury from severe TBI patients which were compared to protein patterns identified in control CSF from individuals undergoing elective neurosurgical procedures. We also examined the relationship between these early proteomic readouts and functional outcomes at six months post-TBI. We hypothesized that this approach would detect CSF proteins associated with neuroinflammation, as well as the identification of novel proteins not previously known to be affected by TBI.

## Materials and methods

### Patient population and sample collection

The study was conducted in accordance with the National Statement on Ethical Conduct in Research Involving Humans of the National Health and Medical Research Council of Australia (NHMRC), following approval by the Alfred Hospital Human Ethics Committee. Sixteen TBI patients (3 females and 13 males) were recruited at the Alfred Hospital, Melbourne, with delayed informed consent obtained from the next of kin (Table 1). Inclusion criteria required a post-resuscitation Glasgow Coma Scale (GCS) score of ≤ 8, indicating severe TBI, and the necessity for an extraventricular drain for intracranial pressure (ICP) monitoring and therapeutic release of CSF (when the ICP was > 20 mmHg). The implantation of extraventricular drains for this purpose is common practice at our institution for patients with severe TBI and raised ICP. Individuals with a history of neurodegenerative diseases, HIV, chronic inflammatory diseases, or pregnancy were excluded.

**Table 1:** Demographic data of TBI and control patients

Variables	Values
TBI	
Age, years, median (range)	30 (21–55)
Gender, *n* (%)	
Males	13 (81%)
Females	3 (19%)
Mechanism of injury, *n* (%)	
Motor vehicle	6 (38%)
Motor bicycle	4 (25%)
Pedestrian	2 (12%)
Penetrating	1 (6%)
Fall/jump	3 (19%)
GCS, median (range)	5 (3–8)
ISS, median (IQR)	37 (17–50)
Marshall score*, median (range)	3 (2–6)
Primary brain injury type, *n* (%)	
Focal	8 (50%)
Diffuse	7 (44%)
Focal and diffuse	1 (6%)
Hypoxia, *n* (%)	6 (38%)
GOSE**, median (range)	4 (1–6)
Unfavorable outcome, *n* (%)	11 (69%)
Favorable outcome, *n* (%)	3 (19%)
Not reported, *n* (%)	2 (12%)
Control	
Age, years, median (range)	70 (38–85)
Gender, *n* (%)	
Males	5 (45%)
Females	6 (55%)

Computer tomogram (CT) scans were performed within 4 h from TBI for injury classification, followed by surgical implantation of an extraventricular drain. Patients were managed in the intensive care unit following our institution’s protocol, as described [20, 21]. Fresh CSF was collected daily over 24 h into a drainage bag on ice, beginning from the day of admission (day 0), within 24 h from the TBI, up to day 10 after injury or when the catheter was removed as part of the patient’s management. CSF was centrifuged at 2000*g* for 15 min at 4 °C, and the supernatant stored at − 80 °C until analysis. Between 8 and 16 CSF samples were available per timepoint (Day 0, 1, 2, 4, 7 and 10 post-injury), with a total of 80 samples from 16 TBI patients available for analysis. The number of samples per day were as follows: Day 0–13; Day 1–15; Day 2–13; Day 4–16; Day 7–15; Day 10–8. Sample n’s were determined by availability of samples in our TBI biobank.

The Glasgow Outcome Scale—Extended (GOSE) was assessed at 6 months post-TBI by phone interview as described previously [22].

### Control patients and sample collection

Control CSF samples were obtained from 11 individuals (6 females and 5 males) undergoing elective neurosurgery for reasons other than a TBI. Ethics approval was granted by the Alfred Hospital Human Ethics Committee, and informed consent was obtained prior to surgery. Exclusion criteria were consistent with that for the TBI cohort, with the addition of no previous TBIs within the past 24 months. Conditions noted for the control patients included insertion of a ventriculoperitoneal shunt (*n* = 5), shunt replacement (*n* = 1), cerebellar and occipital metastatic lesions (*n* = 1), cochlear acoustic neuroma (*n* = 1), cerebral aneurism (*n* = 2) and arachnoid cysts (*n* = 1). Control samples (1 per patient) were processed and stored as described above for the TBI samples (Table 1).

### LC–MS/MS

**Fig. 1:**
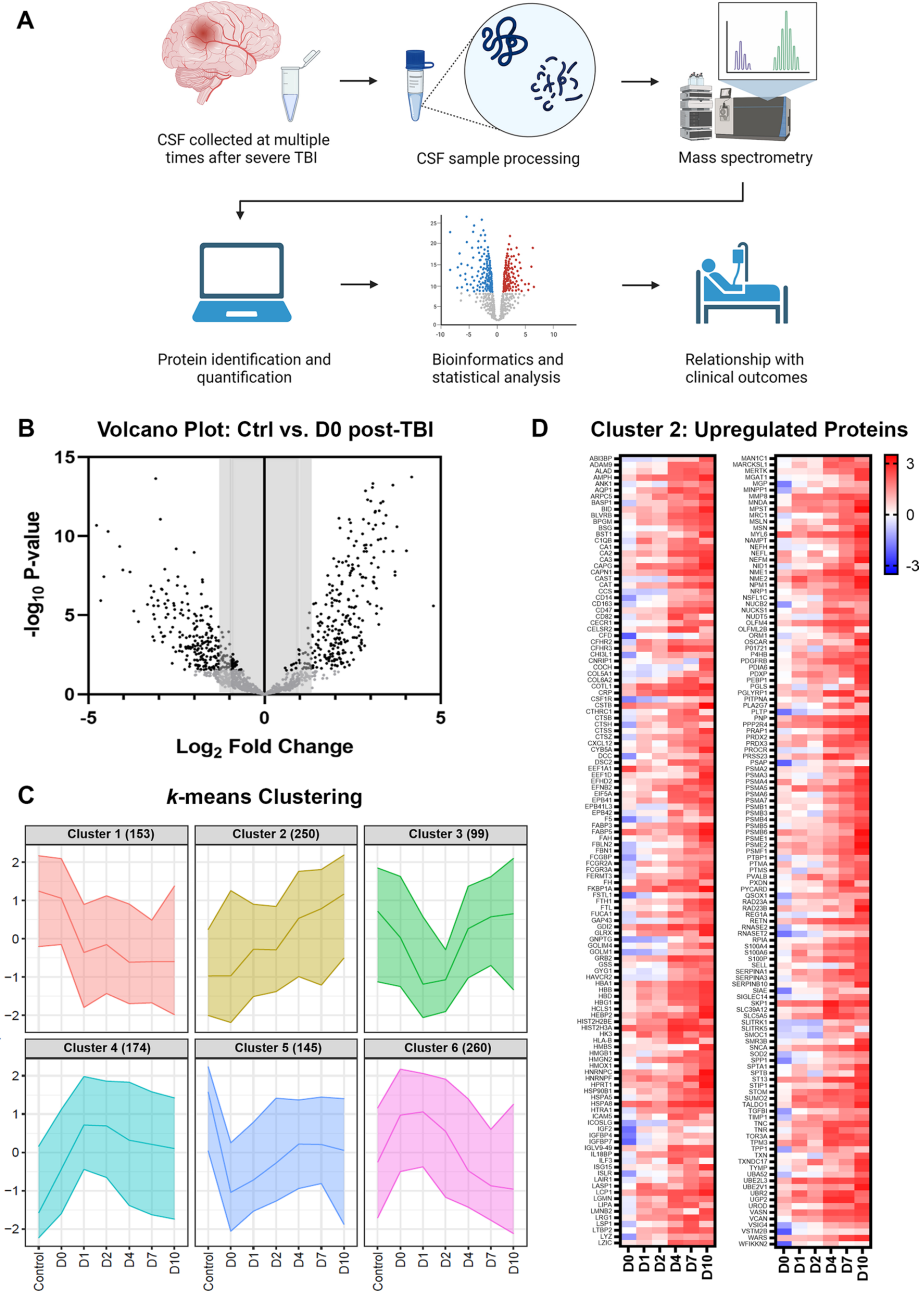
Overview of proteomics analysis. **A** Schematic of overall experimental design. CSF = cerebrospinal fluid; TBI = traumatic brain injury; TMT = tandem mass tag labeling. Created in Biorender.com. **B** Volcano plot demonstrates considerable changes in protein levels between control (Ctrl) and Day 0 (D0) TBI samples. Significantly altered proteins are indicated in black (log_2_ fold change FDR cut-off < 0.05), and non-significant proteins in grey. **C** *k*-means clustering was used to identify 6 distinct temporal patterns of protein expression after TBI. The number of proteins in each cluster is noted in parentheses. **D** Proteins categorized as cluster 2, those with upregulation of log2 fold change over the time course after TBI, compared to control (Ctrl), are presented via heat map

A total of 91 CSF samples collected from TBI patients and controls (80 TBI and 11 controls) were analyzed by quantitative proteomics using tandem mass tag (TMT) labelling (Fig. 1A).

The CSF samples were lysed in 4% SDS, 100 mM Tris, pH8.1, boiled at 95 °C for 10 min and sonicated using a probe sonicator. The samples were prepared for the acquisition by LC MS/MS using the method described previously [23]. The proteins were trapped on S-Trap mini columns (Profiti) and digested to peptides using sequencing grade trypsin with an enzyme to protein ratio of 1:50 and incubated overnight at 37 °C. Using a Pierce Quantitative Colorimetric Peptide Assay Kit (Thermo Scientific), equal tryptic peptide amounts of each sample were labelled with the TMT-11plex reagent set (Thermo Scientific) according to the manufacturer’s instructions. Individual samples were then pooled into multiple plexes, which have been acquired individually by LC–MS/MS to maximum the number of peptide and protein identifications. The LC MS/MS data was acquired using a Dionex UltiMate 3000 RSLCnano for peptide separation and analyzed with an Orbitrap Fusion Tribrid mass spectrometer (ThermoFisher Scientific) for 158 min gradient. The instrument was operated in data-dependent acquisition mode to automatically switch between full scan ms1 (in Orbitrap), ms2 (in ion trap) and ms3 (in Orbitrap) acquisition. Each survey full scan (380–1580 m/z) was acquired with a resolution of 120,000.

Raw data files were analyzed using MaxQuant v1.6.5.0 [24] and its implemented Andromeda search engine [24] against the reference human proteome (Human Swissprot, March 2019) to identify CSF proteins and to obtain quantitative ms3 reporter ion intensities. Carbamidomethylation, N-terminal acetylation, and oxidation of methionine were used as a fixed and variable modifications respectively. TMT reporter ion masses were also used as variable modifications. Approximately 900 proteins were identified per sample. As human albumin was very abundant, as reported previously in the CSF acutely after severe TBI [21], it was excluded from further analysis.

### Statistical analysis

Statistical analysis was performed using various statistical packages in R version 3.6.0 (2019) using an x86_64-pc-linux-gnu platform, or Prism GraphPad v 8.0.2 (2020). Firstly, contaminant proteins, reverse sequences and proteins identified “only by site” were filtered out. In addition, proteins that were identified by a single peptide and proteins not identified/quantified consistently in the same condition were excluded. The protein intensity data was converted to log2 scale, the samples grouped by condition/time, and missing values imputed using the ‘Missing not At Random’ method, which uses random draws from a left-shifted Gaussian distribution of 1.8 standard deviation apart with a width of 0.3. The data was background corrected and normalized by variance stabilizing transformation (vsn; https://bioconductor.org/packages/3.10/bioc/html/vsn.html).

As most samples were from male subjects, consideration of sex as a statistical variable was not possible. However, age was incorporated into the statistical modelling. Protein-wise linear models combined with empirical Bayes statistics were used for the differential expression analyses. The *limma* package [25] from R Bioconductor was used to generate a list of differentially expressed proteins for each pair-wise comparison. A cutoff of the *adjusted p-value* of 0.05 (Benjamini–Hochberg method) along with a log2 fold change of 1 was applied to determine significantly regulated proteins in each pairwise comparison, as visualized via volcano plots and heat maps. An adjusted *p*-value cut-off of 0.05 along with an absolute log2 fold change of at least 1 was applied to determine significantly regulated proteins for each pairwise comparisons. Once normal distribution of data was confirmed for each protein, one-way analysis of variance (ANOVA) was performed to determine potential differences between groups, followed by Dunnett’s multiple comparison tests as post-hoc analysis. Only significant post-hoc analyses are reported graphically (*p* < 0.05) when the ANOVA reported statistical significance overall, with data presented as violin plots to visualize within-group distribution of values. Furthermore, an unsupervised clustering method, *K*-means clustering (*K* = 6), was used on z-transformed data to group proteins with similar expression across different time points. All graphs were generated using R or Prism GraphPad v8.0.2.

An overlap analysis of protein quantified in this study was performed against the published reference CSF proteome for *Homo sapiens*. [26] The “gprofiler2” R package was used to perform functional and pathway enrichment analysis of all differentially expressed proteins, and a subset of upregulated proteins in TBI patients. A hypergeomentric test was performed on above protein-sets against gene ontology (GO) and Reactome (REAC) databases. An adjusted *p*-value (Benjamini–Hochberg) < 0.05 was used to obtain a list of statistically enriched GO terms and pathways.

Finally, logistic regression modelling incorporating age as a continuous variable was used to explore potential relationships between protein levels and patient outcomes, using the GOSE [27]. Outcome was dichotomized as unfavorable (GOSE scores 1–4) or favorable (GOSE scores 5–8). D10 data was not included in this modelling due to low patient numbers (< 10).

### Data availability

Data are available via ProteomeXchange with identifier PXD035289.

## Results

### Patient demographics

We recruited 16 patients aged between 21 and 55 years of age (median age 30 years), with 81% being males (Table 1). Control patients ranged from 38 to 85 years of age (median 70 years), with males making up 45%. The greatest proportion of TBI patients sustained injuries in motor vehicle accidents (*n* = 6; 38%), followed by other mechanisms including motor bicycle and pedestrian accidents, jumps or falls, and penetrating injuries. The GCS at the scene was ≤ 8 for all patients, reflecting severe TBI (median 5; range 3–8). The high injury severity of this cohort is also indicated by the elevated ISS, representing a combination of brain and extracranial injuries (median score 37). Primary brain injury was defined using the CT-based Marshall classification scoring system, where a focal brain injury was defined by the presence of an evacuated lesion or non-evaluated high- or mixed-density mass lesion > 25 ml (*n* = 8; 50%) [21].

16 patients with severe TBI and 11 control patients were recruited to the study. A GCS score ≤ 8 corresponds to severe TBI. Injury Severity Score (ISS) reflects combined cranial and extracranial injuries: 0 = no injury, 75 = maximal untreatable injury. Marshall CT score [28]: I = diffuse injury (no visible pathology on CT scan) to VI (non-evacuated mass lesion). *Not reported for n = 3 patients. The GOSE was assessed at 6 months post-injury: score 1 = dead, 2 = vegetative state, 3 = lower severe disability, 4 = upper severe disability, 5 = lower moderate disability, 6 = upper moderate disability, 7 = lower good recovery, 8 = upper good recovery. GOSE was dichotomized into unfavorable (scores 1–4) or favorable categories (scores 5–8).

### Proteomics overview

We identified a total of 1584 protein sequences across all samples. After applying stringent pre-processing and filtering criteria, 1152 proteins were quantified for downstream statistical analysis. Comparison to a published reference human CSF proteome [26] confirmed that ~ 80% of proteins in our dataset were CSF associated, and > 50% were predicted to be secreted (i.e., contained signal peptide sequences necessary for secretion).

We first performed a hypothesis-free, data-driven exploration of the dataset and found a total of 1083 proteins significantly regulated (up or down) after TBI (at any timepoint) compared to control samples. The proteome detected on D0 showed the greatest difference compared to control samples, as visualized by volcano plot (Fig. 1B).

K-Means cluster analysis was next performed to identify groups of proteins that showed similar temporal patterns of expression after TBI (Fig. 1C). *k* = 6 was chosen as the optimal number of distinct clusters based on the elbow methods utilizing sum of squares information. Six distinct profiles of protein expression, arbitrarily designated Cluster 1 to 6, differentiated between proteins that decrease over time post-injury (Cluster 1; *n* = 153), proteins that increase over time post-injury (Cluster 2; *n* = 250; illustrated by heatmap in Fig. 1D), proteins that decrease acutely on D1 and D2 post-injury then recover to control levels by D7 and D10 (Cluster 3; *n* = 99), proteins that increase acutely on D1 and D2 post-injury, then plateau (Cluster 4; *n* = 174), proteins that decreased very acutely (D0) then recover to control levels (Cluster 5; *n* = 145), and proteins that increase acutely (D0 and D1) then decrease to control levels or below by D7 and D10 (Cluster 6; *n* = 260).

From the full dataset, the top 10 most upregulated proteins per timepoint were extracted (Table 2). This list contains proteins involved in neutrophil functions, including S100 binding protein A8 (S100A8), S100A9, S100A12, myeloperoxidase (MPO), neutrophil elastase (ELANE) and matrix metalloproteinase 8 (MMP8), as well as cathepsin G (CTSG) across the entire time course (D0-D10). For example, ELANE was the second most highly upregulated protein on D1 and the top protein on D2–D10, followed by MPO on D2-D7. Several apolipoproteins including APOC2, APOC3 and APOF were also highly upregulated particularly at acute timepoints (D0-D1), as well as three canonical histone isoforms, HIST2H3A, HIST1H2AJ and HIST2H2BE (on D0 and D4).

**Table 2:** Top 10 upregulated proteins per timepoint

D0	D1	D2	D4	D7	D10
S100A12	MPO	ELANE	ELANE	ELANE	ELANE
HIST2H3A	ELANE	MPO	MPO	MPO	S100P
APOC2	CAMP	CAMP	S100P	S100P	S100A12
APOF	S100A12	S100A12	S100A12	S100A12	PSMB4
FGB	CHI3L2	CTSG	CAMP	MMP8	CTSG
APOC3	CTSG	CHI3L2	STOM	PSMB4	HCLS1
FGG	MMP8	S100A8	HIST2H2BE	CTSG	MPO
PLEK	S100A8	S100A9	CTSG	CAMP	MMP8
GCA	S100A9	MMP8	HIST2H3A	S100A9	GLRX
HIST1H2AJ	FCGR3B	FCGR3B	MMP8	STOM	S100A9

*APOC2, APOC3 and APOF* Apolipoproteins; *CAMP* Cathelicidin antimicrobial peptide; *CHI3L2* Chitinase-3-Like Protein 2; *CTSG* Cathepsin G; *D* day post-injury; *ELANE* Neutrophil elastase; *FCGR3B* Fc Gamma Receptor IIIb; *FGB* Fibrinogen beta chain; *FGG* Fibrinogen gamma chain; *GCA* Grancalcin; *GLRX* Glutaredoxin; *HCLS1* Hematopoietic Cell-Specific Lyn Substrate 1; *HIST1H2AJ, HIST2H2BE and HIST2H3A* Histone isoforms; *MMP8* Matrix metalloproteinase 8; *MPO* Myeloperoxidase; *PLEK* Pleckstrin; *PSMB4* Proteasome subunit beta type-4; *STOM* Stromatin; *S100P, S100A8, S100A9 and S100A12* S100 calcium binding protein

### Functional enrichment analysis

**Fig. 2:**
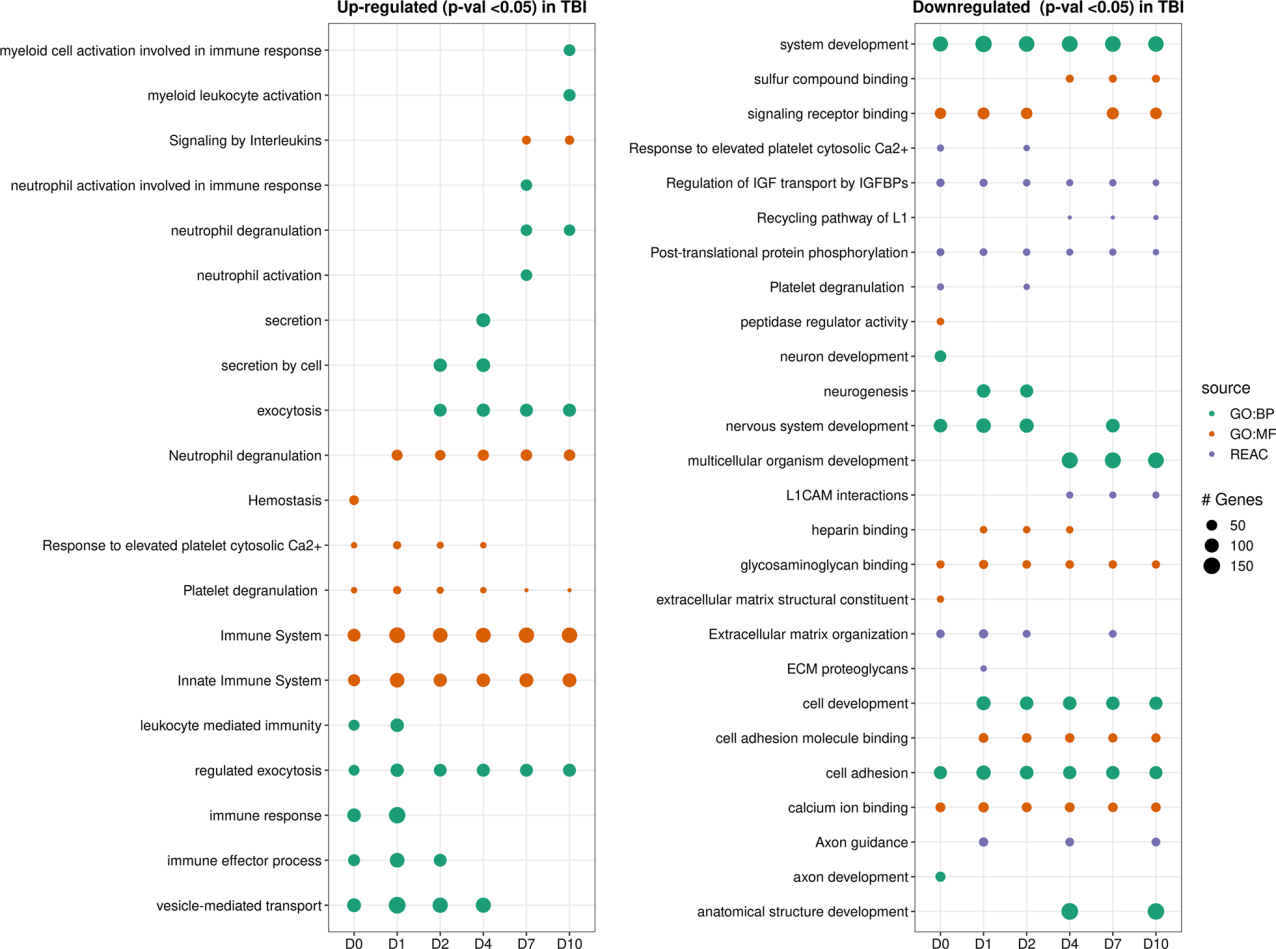
Pathway enrichment analysis of TBI. Top 5 most highly up-regulated and down-regulated pathways (based on lowest enrichment FDR-adjusted p-values) in CSF from patients with severe TBI compared to controls, by timepoint (D0 = day 0, D1 = day 1 post-injury, etc.). FDR = false discovery rate; *p-*value cut-off of < 0.05. GO:BP = gene ontology biological process (red); REAC = reactome database (blue). IGFBPs = Regulation of insulin-like growth factor (IGF) transport and uptake by insulin-like growth factor binding proteins. The size of the bubble for each pathway and timepoint represents the number of proteins included in the pathway. No GO-MF (gene ontology molecular functions) are reported in **A** as none were significantly upregulated in TBI samples compared to controls

Functional enrichment analysis was performed to identify the biological pathways most affected by TBI. Numerous gene ontology biological processes (GO-BP), molecular functions (GO-MF) and reactome pathways (REAC) were identified as significantly and differentially expressed in CSF from TBI patients, against a background proteome dataset previously reported background in *Homo sapiens* CSF [26]. We identified the top 5 significantly differentially expressed terms for each timepoint (*p* < 0.05; lowest enrichment FDR-adjusted *p-*values), for a total of 20 terms upregulated (Fig. 2A) and 27 terms downregulated (Fig. 2B) compared to controls. Some terms were common across multiple or all timepoints, while others were timepoint specific.

The most highly upregulated pathways included proteins involved in innate immune responses (e.g., myeloid cells and neutrophils), interleukin signaling, and proteins regulating exocytosis. Several pathways mediating neutrophil recruitment, activation, and degranulation were upregulated across the time course, in agreement with the most highly upregulated proteins in our dataset (Table 2). In particular, neutrophil degranulation was extracted as a key upregulated pathway in both the Gene ontology (GO-BP) and Reactome (REAC) databases. In contrast, the most robustly downregulated proteins in CSF from TBI patients compared to controls were typically those involved in nervous system, axonal, and neuronal development, as well as axonal guidance, neurogenesis, and cell adhesion.

### Differential regulation of known proteins of interest

**Fig. 3:**
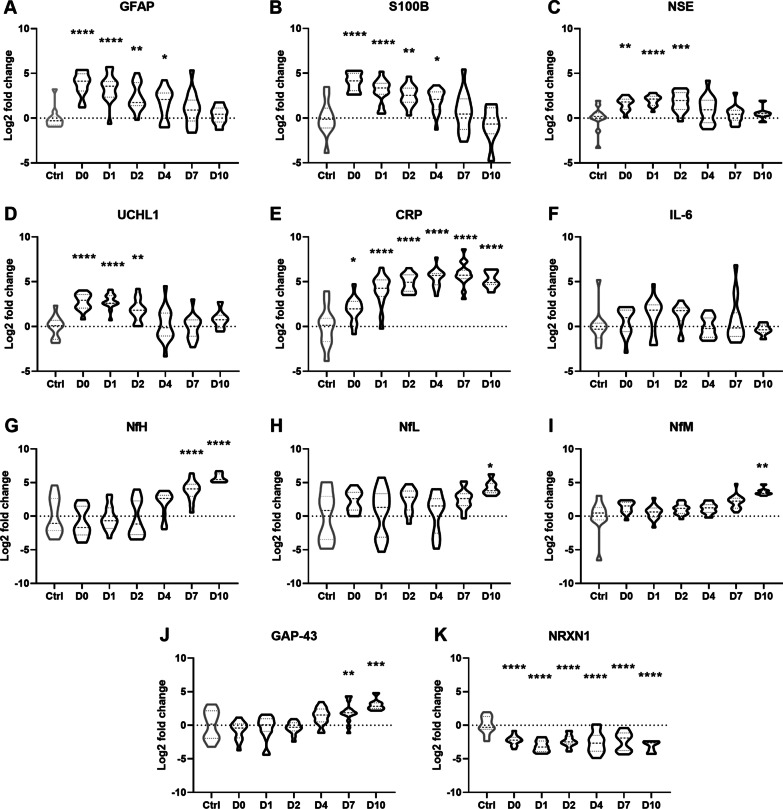
Detection of established injury biomarkers in CSF after severe TBI. **A**–**F** Temporal changes in CSF protein levels after TBI compared to controls, of GFAP (glial fibrillary acidic protein), S100B (S100 calcium binding protein B), NSE (neuron specific enolase), UCHL1 (ubiquitin C-terminal hydrolase L1), CRP (C-reactive protein) and IL-6 (interleukin-6). **G**–**K** Several biomarkers of neuronal and axonal damage were also detected, including neurofilament (Nf) proteins—heavy (H), light (L) and medium (M) chains; GAP-43 (growth-associated protein-43) (**D**); and NRXN1 (neurexin-1). Group *n*’s = 11 (ctrl), 13 (day 0), 15 (day 1), 13 (day 2), 16 (day 4), 15 (day 7) and 8 (day 10). One-way ANOVAs with Dunnett’s multiple comparison tests; *p < 0.05, **p < 0.01, ***p < 0.001, ****p < 0.0001. Dotted line = 0 fold change relative to controls

Several well-known proteins that have been previously explored for their biomarker potential after TBI were identified in our cluster analysis as either cluster 2, 4 or 6 [7, 11, 12, 29, 30]. The astrocytic markers glial fibrillary acidic protein (GFAP) and S100B peaked on D0 post-injury, remaining elevated to D4, before returning to control levels by D7 and D10 (Fig. 3A, B). Neuron specific enolase (NSE) and ubiquitin C-terminal hydrolase L1 (UCHL1) showed a similar temporal profile, peaking on D0–D2 post-injury (Fig. 3C, D), likely reflecting primary neuronal damage. The inflammatory marker C-reactive protein (CRP) increased across the time course, peaking between D4 and D7, and remained significantly elevated compared to control levels on D10 (Fig. 3E). By contrast, the cytokine interleukin-6 (IL-6) was not significantly altered across the time course (Fig. 3F).

The neurofilament components NfH, NfL, and NfM, indicative of neuronal and/or axonal damage [31, 32], were elevated on D7 and/or D10 post-injury (Fig. 3G–I). Growth-associated protein 43 (GAP-43) followed a similar pattern to the neurofilaments, with a delayed increase on D7-D10 post-injury (Fig. 3J). Finally, the synaptic protein neurexin-1 (NRXN1), a potential biomarker of neurodegeneration in Alzheimer’s disease, was found to be consistently decreased after TBI compared to control (Fig. 3K).

**Fig. 4:**
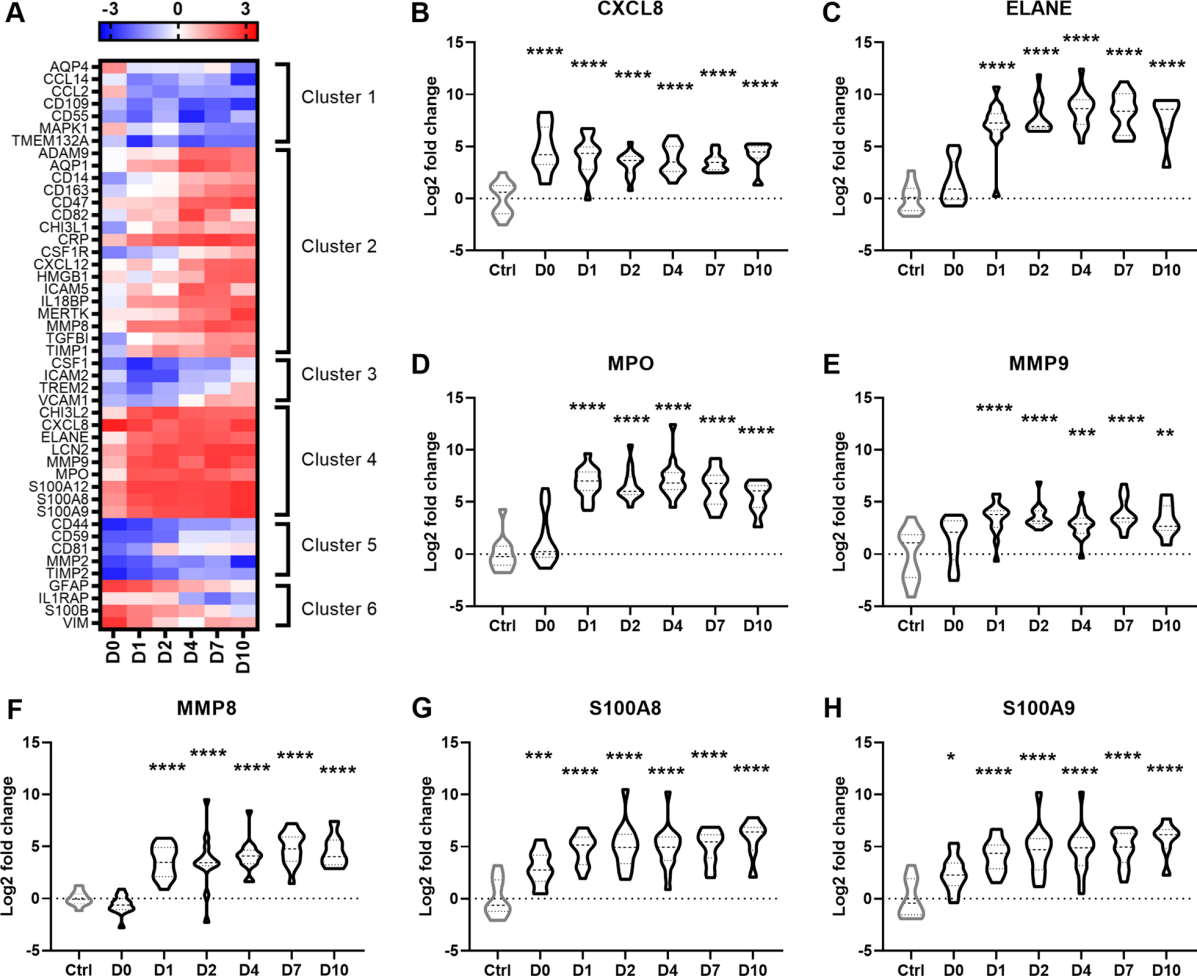
Selected immune- and inflammation-related proteins altered by severe TBI. **A** Heat map represents log2 fold change normalized to the average of the control group. **B**–**H** Several inflammation-related proteins identified in cluster 2 are involved in neutrophil mobilization, recruitment and activation, including CXCL8 (interleukin-8), ELANE (neutrophil elastase), MPO (myeloperoxidase), MMP9 (matrix metalloproteinase-9) and MMP8, S100A8 (S100 calcium binding protein A8) and S100A9. Group *n*’s = 11 (ctrl), 13 (day 0), 15 (day 1), 13 (day 2), 16 (day 4), 15 (day 7) and 8 (day 10). One-way ANOVAs with Dunnett’s multiple comparison tests; *p < 0.05, **p < 0.01, ***p < 0.001, ****p < 0.0001. Dotted line = no fold change relative to controls

Several other proteins involved in innate immune responses were also identified. Focusing in on an a-priori list of key immune and inflammation-related proteins either known or suspected to be altered in the CSF by severe TBI (Fig. 4A), we noted that while several immune-related proteins were downregulated (e.g., CD44 and CD55), the majority of inflammatory mediators showed a pattern of elevation consistent with *k*-means clusters 2 and 4 (Fig. 4A). Cluster 2 in particular contained numerous proteins involved in neutrophil mobilization, recruitment and activation. The chemotactic cytokine interleukin-8 (CXCL8) was robustly increased after TBI regardless of timepoint (Fig. 4B). ELANE and MPO, typically released by activated polymorphonuclear leukocytes upon degranulation, were elevated from D1 onwards (Fig. 4C, D). The metalloproteinases MMP9 and MMP8 were similarly elevated from D1 across the time course (Fig. 4E, F), while pro-inflammatory cytosolic proteins S100A8 and S100A9 (calprotectin), abundantly expressed in neutrophils and involved in neutrophil recruitment, adhesion and migration, were also consistently upregulated to at least D10 after severe TBI (Fig. 4G, H). As noted above, ELANE, MPO, MMP8 and the S100A proteins (S100A8, S100A9 and S100A12; the latter not shown) were among the most highly upregulated proteins in TBI samples compared to controls, at 5–10 log2 fold change.

### Identification of novel downregulated proteins

**Fig. 5:**
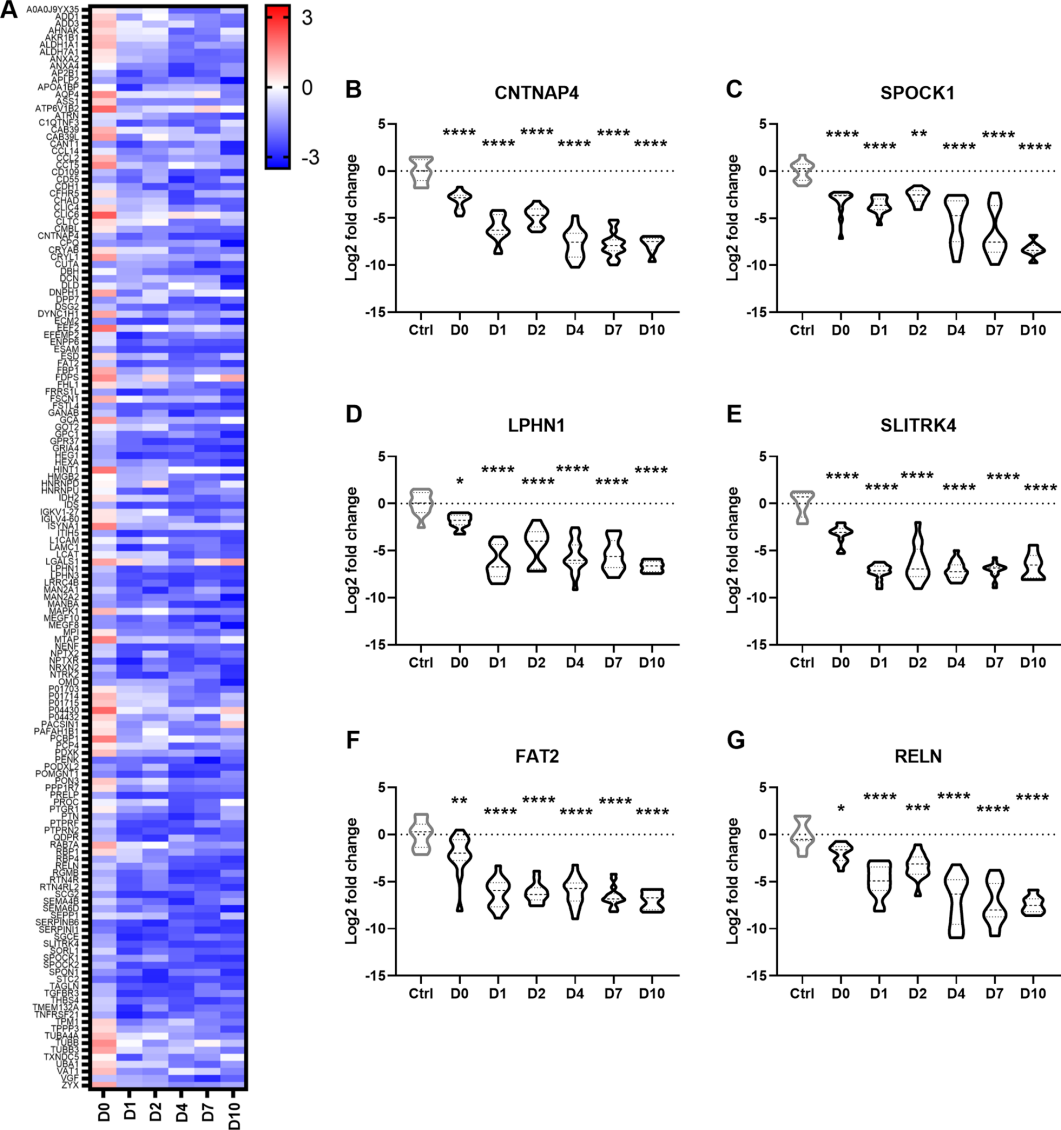
Significantly downregulated proteins in CSF after TBI compared to controls. **A** Heat map depicts proteins in Cluster 2, those that showed downregulation (log2 fold change) over the time course after TBI compared to control (Ctrl). **B**–**G** Some of the most downregulated proteins included CNTNAP4 (contactin associated protein family member 4), SPOCK1 (testican-1), LPHN1 (latrophilin 1), SLITRK4 (SLIT And NTRK Like Family Member 4), FAT2 (FAT Atypical Cadherin 2) and RELN (Reelin). Group *n*’s = 11 (ctrl), 13 (day 0), 15 (day 1), 13 (day 2), 16 (day 4), 15 (day 7) and 8 (day 10). One-way ANOVAs with Dunnett’s multiple comparison tests; *p < 0.05, **p < 0.01, ***p < 0.001, ****p < 0.0001. Dotted line = 0 fold change relative to controls

Building upon the identification of downregulated protein pathways (Fig. 2B), and *k*-means clustering (Fig. 1C), we focused on specific proteins that were considerably lower in CSF from TBI patients compared to controls, many of which have not been previously considered in the context of TBI. Cluster 1 is comprised of 153 proteins, which were stable or slightly elevated on D0 and then reduced up to D10 relative to controls (Fig. 5A). Among the most significantly downregulated proteins, particularly at D7 and D10, was CNTNAP4 (contactin associated protein family member 4), a presynaptic protein involved in dopaminergic and GABAergic neurotransmission [33], and SPOCK1 (also known as testican-1), a highly conserved proteoglycan that is typically expressed in the thalamus and cerebellum, which was recently identified as a potential biomarker for sepsis [34] (Fig. 5B, C). Both LPHN1 (latrophilin 1), a G protein-coupled receptor involved in cell adhesion and signal transduction implicated in neurotransmitter release and control of presynaptic calcium [35], and SLITRK4 (SLIT And NTRK Like Family Member 4), a poorly-characterized transmembrane protein with related family members implicated in neurite outgrowth and neuronal survival [36], were among the most significantly downregulated proteins from D1 to D10 post-injury compared to controls (Fig. 5D, E).

Finally, the protocadherin FAT2, a critical cell adhesion molecule in cerebellum development and recently recognized as a CSF biomarker for Parkinsonism [37], was another of the most downregulated proteins between D1 and D10 post-injury, alongside Reelin (RELN), a major regulator of mammalian brain development, dendrite outgrowth and synaptic plasticity in the adult brain (Fig. 5F, G). [38]

### Protein changes associated with patient outcomes

Outcome at 6 months post-injury was generally poor for this cohort of severe TBI patients, with a median GOSE of 4 (range of 1–6). Most patients (69%; *n* = 11) had an unfavorable GOSE (score of 1–4), while no patients had a GOSE of either 7 or 8. GOSE scores were dichotomized to unfavorable (1–4) and favorable (5–8) outcomes. Logistic regression analysis for each protein was performed for individual timepoints considering protein expression as a main variable, with or without incorporating age as a covariate. The analysis detected a limited subset of seven proteins found to be associated with GOSE outcome using a significance cut-off level of *p* < 0.05. These included Ganglioside GM2 activator (GM2A) on D0, Calsyntenin 1 (CLSTN1), FAT Atypical Cadherin 2 (FAT2), Glucosidase II Alpha Subunit (GANAB), Lumican (LUM) and neuronal pentraxin-1 (NPTX1) on D2, and Secreted Frizzled Related Protein 2 (SFRP2) on D4 (Table 3).

**Table 3:** Proteins significantly correlated with GOSE outcome scores

Protein	Timepoint	Log2 Fold change vs. Control	Log odds	*P*-value
GM2A	D0	3.51	− 3.67046E+15	< 2e−16
CLSTN1	D2	2.73	− 2.827E+15	< 2e−16
FAT2	D2	6.62	3.26394E+15	< 2e−16
GANAB	D2	2.09	1.06114E+15	< 2e−16
LUM	D2	− 0.851	− 5.7196E+15	< 2e−16
NPTX1	D2	2.9	− 3.5895E+15	< 2e−16
SFRP2	D4	− 2.45	− 2.33829E+15	< 2e−16

*CLSTN1* Calsyntenin 1; *D* day post-injury; *FAT2* FAT Atypical Cadherin 2; *GM2A* Ganglioside GM2 activator; *GANAB* Glucosidase II Alpha Subunit; Lumican (LUM); *NPTX1* neuronal pentraxin-1; *SFRP2* Secreted Frizzled Related Protein 2

## Discussion

Further elucidation of the pathophysiology of severe TBI is urgently needed in order to discover diagnostic and prognostic biomarkers as well as therapeutic targets to improve patient outcomes. This study examined protein changes in CSF samples collected longitudinally from severe TBI patients for up to 10 days post-injury in an unbiased exploratory analysis of the CSF proteome, aiming to identify novel protein biomarkers and pathways affected by injury. The greatest proteome difference between the TBI and control samples occurred within the first 24 h of injury (= D0). Cluster analysis identified six distinct groups of proteins that had similar temporal patterns after TBI (i.e., decrease or increase over time, initial decrease with recovery by D7 and D10, initial increase before plateauing, acute decrease with rapid recovery, and acute increase before drop to control levels or lower). Although our study did not validate the findings with alternative techniques (e.g. enzyme-linked immunoassays), the temporal profiles of several well-known proteins (e.g., GFAP, S100B, NSE, UCHL1, NfL, CRP) after TBI are similar to those previously published [11, 12, 29, 30, 39, 40] which provides validation to the overall findings and methodology. A particular strength of this study, and a testament to our hypothesis-free, data-driven approach, was the discovery of several new protein alterations that have not been previously identified in the pathophysiological aftermath of severe TBI (e.g., S100A8, S100A9, S100A12, and CTSG). Furthermore, the longitudinal nature of the study allowed for the characterization of unique temporal trajectories of these proteins beyond the more commonly studied acute timepoints [41], and how they related to recovery at 6 months post-injury.

### Novel protein changes in the CSF after severe TBI

S100A8, S100A9, S100A12, and CTSG are all implicated in neutrophil function [42, 43], with recent reports showing that S100A8 and S100A9 are associated with neuroinflammation in preclinical models of TBI [44, 45]. A clinical study exploring the prognostic potential of S100A12 in severe TBI, found that its serum levels were significantly elevated compared to controls alongside other markers (e.g., S100B, CRP), and were also independently associated with mortality and adverse events [46].

Elevated S100A8, S100A9, S100A12 and CTSG was observed in association with several other neutrophil-related proteins (e.g., MPO, ELANE, MMP8) being amongst the most highly upregulated, supporting established evidence that neutrophils are critical mediators of acute TBI pathophysiology [47, 47, 48, 48–52]. Conversely, we also found that many neutrophil-associated proteins, including S100A9, S100A12, and CTSG, were upregulated at later timepoints, suggesting that their influence may extend into the sub-acute phase, which has important therapeutic implications for a delayed intervention to modulate inflammatory processes after TBI. Our pathway enrichment analysis also supports the fact that neutrophil- and immune system-related pathways are among the top five most highly upregulated at each timepoint after TBI.

In our study, we also identified proteins that were downregulated in the TBI CSF samples. Among the top 10 proteins found at each recovery time are some that have not been previously reported to be altered after clinical TBI (i.e., CNTNAP4, SPOCK1, RELN, FAT2, LPHN1, SLITRK4). Many of these proteins have been implicated in neural development, which is consistent with our pathway analysis, including nervous system development, axon development, neuron development, neuron projection development, and/or neurogenesis being among the top 5 downregulated pathways at each timepoint. These proteins also hold the potential as biomarkers of brain injury and pharmacological targets. The downregulation of CNTNAP4 has been demonstrated in several neurological conditions, including temporal lobe epilepsy [33] and major depressive disorder [53]. Specifically, CNTNAP4 is decreased in the brains of both epileptic patients and mice: Knock-down of CNTNAP4 in mice increased epilepsy susceptibility, while its overexpression decreased epileptic behavior [33]. This may have important implications in the context of severe TBI where the later development of acquired epilepsy is relatively common [52, 54, 55].

Several proteome-wide studies have reported that CSF levels of SPOCK1/testican-1 are reduced in Alzheimer’s patients [56, 57]. Recent preclinical findings have shown that RELN is decreased in the thalamus and hippocampus following experimental TBI [58], while in an in vitro model of glutamate-induced excitotoxicity, RELN protected hippocampal neurons [58]. Of relevance to the consequences of TBI, hippocampal reelin infusions mitigated depression and cognitive deficits alongside increased hippocampal neurogenesis in a rat model of chronic stress [59]. Finally, consistent with our observation of reduced FAT2 levels in CSF being associated with GOSE scores at 6 months post-injury, a previous study reported that gene expression of FAT2 was downregulated in the cerebellum of mice with neurodegenerative ataxia [60].

### Relationship between CSF proteins and long-term outcome

There is an urgent need for novel and valid biomarkers capable of predicting long-term outcomes in severe TBI patients. For this purpose, we investigated whether the proteins detected in CSF were correlated to GOSE scores at 6 months post-injury. GM2A (D0), CLSTN1 (D2), FAT2 (D2), GANAB (D2), LUM (D2), NPTX1 (D2), and SFRP2 (D4) were all found to be statistically associated with the GOSE score. Although these findings are novel in the context of TBI, however several of these proteins are recognized as promising biomarkers in other neurodegenerative conditions. For instance, GM2A, a lipid transfer protein, has reduced reduced concentrations in the CSF of Parkinson’s patients [61]. CLSTN1, a synaptic protein, is decreased in the CSF of preclinical Alzheimer’s patients before the onset of clinical symptoms and the appearance of neurodegenerative markers [62]. In addition, lower CLSTN1 CSF levels have been linked to frontotemporal dementia-related synapse degeneration [63]. Finally NPTX1, a protein involved in synaptic plasticity, was found in lower concentrations in the CSF of patients with Alzheimer’s disease [64], as well as in patients with symptomatic genetic frontotemporal dementia [65]. Taken together, our initial findings on protein changes in response to TBI in this small patient group warrant further investigation in larger cohorts to ascertain whether these CSF proteins can be used as prognostic biomarkers to identify those TBI patients at risk for poor outcome, as well as functioning as potential pharmacological targets to attenuate those pathways that may modulate secondary brain damage.

## Limitations and future directions

There are some limitations that should be considered for the interpretation of these findings. The TBI cohort of this study is small, and the samples were not available from every patient at each timepoint, which limited the statistical power of GOSE correlative analyses. Further, most TBI participants were male, which prevented us from assessing possible sex differences in the protein analysis. Another limitation related to the TBI versus non-TBI comparison is that the CSF collection procedures varied between the groups. For example, the CSF from the TBI group was collected via a continuous extraventricular drain, which would have pooled between collection times, whereas the non-TBI group involved a more acute CSF collection period (e.g., via ventriculo-peritoneal shunt) and less pooling time. A difference in pooling times between the groups could have influence protein concentrations (e.g., more protein degradation with increased pooling time). There was also an age discrepancy between the TBI patients and the older control group, and limited studies have suggested that the CSF proteome undergoes age-related changes [66, 67]. While we cannot rule out the possibility that age or CSF collection protocol differences between control and TBI samples may account for some of our findings, this potential confound would not be influencing the changes that we observed across the time course after TBI.

It is also important to note that non-TBI control CSF samples are challenging to acquire, especially in terms of obtaining CSF from ‘normal’ healthy patients. Of note, we observed significant differences in the CSF proteome over time after TBI compared to controls, even with a fairly heterogeneous control group. Our findings here mirror those of previous studies that have similarly reported consistently low protein levels regardless of whether control CSF is obtained via ventriculo-peritoneal shunt or lumbar puncture. Nonetheless, large-scale studies that include a more balanced gender ratio with age-matched controls and consistent CSF collection protocols would be useful to both validate and expand our findings. Future studies could also incorporate paired CSF and blood protein measurements for improved capacity to accurately examine the relationship between biomarkers in both the CNS and peripheral compartments.

In conclusion, our proteomic analyses derived from CSF collected for up to 10 days after severe TBI identified several novel proteins and pathway alterations that have not been previously reported in the context of TBI. Neutrophil-related proteins such as S100A8, S100A9, S100A12, and CTSG, involved in innate immune pathways, were among the most highly upregulated, whereas several proteins mediating nervous system development (i.e., SPOCK1, LPHN1, SLITRK4, CNTNAP4, FAT2) were greatly downregulated. Furthermore, we demonstrated a prognostic potential for the CSF proteins GM2A, CLSTN1, FAT2, GANAB, LUM, NPTX1, and SFRP2 based on their significant correlations with the GOSE scores at 6 months post-injury. These novel findings provide a foundation for future biomarker and pharmacological studies that may ultimately improve the clinical management and outcome of TBI patients.

## Data Availability

Data are available via ProteomeXchange with identifier PXD035289.

## Abbreviations

ANOVA: Analysis of Variance
APO2: Apolipoprotein 2
CLSTN1: Calsyntenin 1
CNTNAP4: Contactin associated protein family member 4
CRP: C-reactive protein
CSF: Cerebrospinal fluid
CT: Computed tomography
CTSG: Cathepsin G
CXCL8: CXC chemokine ligand 8 (= interleukin 8)
ELANE: Neutrophil elastase
FAT2: FAT Atypical Cadherin 2
GANAB: Glucosidase II Alpha Subunit
GCS: Glasgow Coma Scale
GFAP: Glial fibrillary acidic protein
GM2A: Ganglioside GM2 activator
GO-BP: Gene ontology, biological process
GO-MF: Gene ontology, molecular function
GOSE: Glasgow Outcome Scale—Extended
HIST2H3A: Histone isoform
ICP: Intracranial pressure
LC–MS/MS: High-resolution mass spectrometry
LPHN1: Latrophilin 1
MMP: Matrix metalloproteinase
MPO: Myeloperoxidase
NfL: Neurofilament light chain
NHMRC: National Health and Medical Research Council
NPTX1: Neuronal pentraxin-1
NSE: Neuron specific enolase
NRXN1: Neurexin-1
REAC: Reactome
RELN: Reelin
S100A8: S100 binding protein A8
SFRP2: Secreted Frizzled Related Protein 2
SLITRK4: SLIT And NTRK Like Family Member 4
SPOCK1: Testican-1
TBI: Traumatic brain injury
TMT: Tandem mass tag
